# An Efficient and Effective Design of InP Nanowires for Maximal Solar Energy Harvesting

**DOI:** 10.1186/s11671-017-2354-8

**Published:** 2017-11-25

**Authors:** Dan Wu, Xiaohong Tang, Kai Wang, Zhubing He, Xianqiang Li

**Affiliations:** 1 OPTIMUS, Photonics Centre of Excellence, School of Electrical and Electronic Engineering, Nanyang Technological University, 50 Nanyang Avenue, Singapore, 639798 Singapore; 2 Department of Electrical and Electronic Engineering, Southern University of Science and Technology, 1088 Xueyuan Avenue, 518055 Shenzhen, People’s Republic of China; 3 Department of Materials Science and Engineering, Southern University of Science and Technology, 1088 Xueyuan Avenue, 518055 Shenzhen, People’s Republic of China

**Keywords:** Computational modeling, III–V semiconductor materials, Photovoltaic cells

## Abstract

Solar cells based on subwavelength-dimensions semiconductor nanowire (NW) arrays promise a comparable or better performance than their planar counterparts by taking the advantages of strong light coupling and light trapping. In this paper, we present an accurate and time-saving analytical design for optimal geometrical parameters of vertically aligned InP NWs for maximal solar energy absorption. Short-circuit current densities are calculated for each NW array with different geometrical dimensions under solar illumination. Optimal geometrical dimensions are quantitatively presented for single, double, and multiple diameters of the NW arrays arranged both squarely and hexagonal achieving the maximal short-circuit current density of 33.13 mA/cm^2^. At the same time, intensive finite-difference time-domain numerical simulations are performed to investigate the same NW arrays for the highest light absorption. Compared with time-consuming simulations and experimental results, the predicted maximal short-circuit current densities have tolerances of below 2.2% for all cases. These results unambiguously demonstrate that this analytical method provides a fast and accurate route to guide high performance InP NW-based solar cell design.

## Background

For future generation solar cells, semiconductor nanowire (NW) arrays have unraveled a new pathway to greatly reduce material consumption and fabrication cost while maintaining or even improving the device performance as compared with their thin film or bulk counterparts [1, 2]. This fascinating feature is largely attributed to the remarkable optical properties of the NWs, including increased absorption [3, 4] and spectral selectivity [5–7]. Among various III–V materials, InP NW arrays have attracted intensive research effort for solar cell application due to the direct bandgap and low intrinsic surface recombination velocity [8]. Up to date, the highest energy conversion efficiency achieved 13.8% for InP NW arrays in a cell of 1 mm^2^ in area [9].

Since the optical properties of NW arrays can be distinctively adjusted by tuning their three-dimensional geometry, to further improve the performance of NW-based solar cells, great attention has been put on how to optimize the morphology and topology of III–V NW arrays to maximize the light absorption [5, 9–13]. Specifically, the NWs’ diameter, periodicity, and arrangement have been investigated to maximize the absorption of solar energy [6, 14–16]. It is reported that tuning the diameter of the NW will change the optical modes existed within the NW. This will lead to localized light absorption maxima for those incident wavelengths corresponding to the respective resonant modes [5, 6, 17, 18]. Also, NW arrays with optimized periodicity or filling ratio (FR) can suppress the reflection and transmission while enhancing the scattering to the incident light resulting in the prolonged optical path and thus the enhanced light absorption [19–21]. Besides, Martin Foldyna et al. have concluded that the dependence of the light absorption on the arrangement of the NW arrays is rather small since the light trapping effect of NWs is based on the individual waveguiding when the light coupling among neighboring NWs is neglected [22].

To find the maximal solar energy harvesting, the effect of the three-dimensional parameters and the arrangement of the NW arrays should be considered together. However, most of the reported optimal geometrical dimensions and arrangement of NW arrays for maximal solar spectrum harvesting are still parameter-space-determinated local optima. Besides, the incident solar spectrum combining with material dispersive properties add more difficulty to analytically solve this problem. Therefore, intensive and time-consuming numerical simulations such as finite-difference time-domain (FDTD) are frequently adopted to address this multi-parameters optimization problem. Sturmberg et al. reported a semi-analytic method to narrow down the range of the optimal dimensions of single diameter NW arrays [13]. Although this method is applicable for various materials, FDTD simulations should still be accompanied to find the exact optimal values. Moreover, this method is less helpful for superb absorber combined with multi-radii NW arrays [23].

In this paper, we present an analytical design for optimal geometrical dimensions of single, double, and multiple diameter InP NW arrays to maximize solar energy absorption. Diameters of NWs are determined by leaky mode resonance and Mie theory whereas the periodicities are identified by construction of an effective medium layer to minimize light reflection and transmission. Squarely and hexagonal distributed NW arrays are both considered. Moreover, intensive FDTD simulations are accompanied to verify the effectiveness of our method. The well matching of the largest short-circuit current densities generated from the NW arrays with the calculated geometrical parameters and the values obtained from FDTD simulations prove the effectiveness of the proposed method to guide the practical NW-based photovoltaic cells design.

## Design for Maximal Light Harvesting of InP NWs

**Fig. 1:**
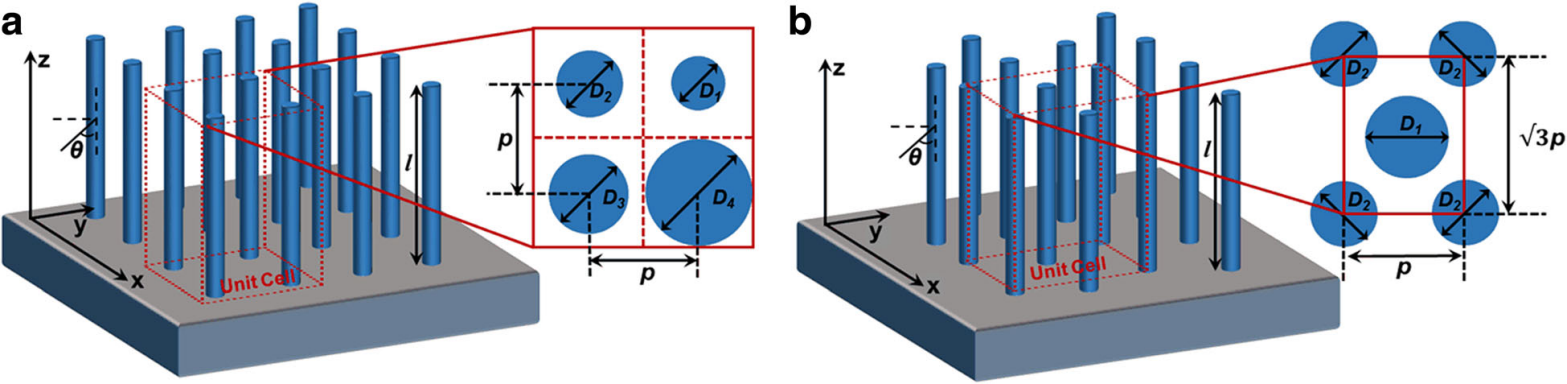
Schematics of vertically aligned InP NW arrays. **a** Squarely and **b** hexagonal NW arrays with insets explaining their respective unit cells

Vertically aligned InP NW arrays are placed upon a semi-infinite SiO_2_ substrate as schematically shown in Fig. 1 with either squarely or hexagonal arrangement. Repeatable unit cells in Fig. 1a, b insets explain respective characterization dimensions for each arrangement. This morphology and topology of the NW arrays are in accord with the majority of the InP NW-based solar cell structures [11, 12, 23, 24]. Within each of the unit cells, the NWs have the same or different diameters as *D*_*i*_. Periodicity *p* is defined as the center to center distance of a pair of adjacent NWs which has the same value for squarely arranged NWs whereas different values for hexagonal NW arrays. Accordingly, the FR of the squarely arranged NW arrays is defined as $\pi {\sum}_{\mathrm{i}=1}^4{D_i}^2/{(4p)}^2$ having the maximal value of *π/4* when the NWs take up the largest volume percentage of the unit cell [25]. Similarly, the FR for hexagonal NW arrays is defined as $\pi {\sum}_{\mathrm{i}=1}^2{D_i}^2/\left(4\sqrt{3}{p}^2\right)$ with the maximal value of $\pi \sqrt{3}/6$ [22]. The length *l* of the NW is set as 2 μm for all cases since they are long enough to absorb more than 90% of the incident energy with proper design [26].

In order to analytically determine each geometrical parameter of NW arrays, the multiple-parameter optimization problem for maximal light harvesting is decomposed into two processes: (1) NWs’ diameter-determinant resonant mode control and (2) FR-affected minimal reflectance and transmittance of incident solar energy. We construct the relationship of individual geometrical parameter with respective determinant process and identify each optimal value leading to maximal light absorption. Double diameter NW arrays are chosen as the design example for illustration of the proposed method. Optimal geometrical dimensions of single diameter NW arrays as a simpler case can also be acquired during the derivation. The diameter and periodicity for four diameter NW arrays can also be calculated as an extension of the example. For squarely arranged double diameters NW arrays, the diameters of the diagonal NWs have the same value as *D*_major_ and the diameters of the rest two NWs are named as *D*_supplementary_. For hexagonal arranged NW arrays, the diameter of the center NW is *D*_major_ and the diameters of the NWs at the peripheral are *D*_supplementary_.

It is reported that NW arrays can support leaky/guided resonance modes, each of which lead to strong absorption peaks. Besides, the fundamental nature of waveguide suggests that the mode number grows with the rise of the diameter of NW. Consequently, the optimal diameter of NW should be large enough to support more modes so as to include larger number of absorption resonances. However, too large diameters of NWs are less preferable since the higher order modes they supported possess more nodes which couple less efficiently to the incident plane waves [13]. Besides, the material property and the incident solar spectrum place other limitations on the selection of the optimal diameter. Only when the resonant modes lie within the absorption region, they can contribute to the photocurrent. The absorption region is defined by the superposition of the material absorbing range of up to critical wavelength and the incident AM 1.5G spectrum [27].

As a result, to quantitatively determine the *D*_major_ of the NW arrays, leaky mode resonance is initially adopted to calculate the respective resonant wavelengths for different diameters of NWs [2]. This gives the distribution of the resonant modes in the absorption region. Therefore, the optimal *D*_major_ should support two modes to satisfy all of the above criterions. Secondly, Mie theory is adopted to calculate the normalized absorption efficiencies of those NWs in step one. Strictly speaking, Mie theory cannot be applied to the situation when the incident wave vector aligned perfectly parallel to the axis of NWs since the eigenvalue equation is ill-defined [28]. However, this situation can be approximated as the glazing incident of incoming light (very small incident angle *θ* with respect to the axis of NW) since at the interface of NW arrays, the wave front of the incident light will be perturbed by the high index of NWs which introduces transverse components to the wave vector allowing the adoption of Mie theory [18]. Therefore, the optimal *D*_major_ are the one who support two modes while keeping the full width at half maximum (FWHM) of the lowest resonant mode in the normalized absorption efficiency spectrum within the absorption region. After acquisition of *D*_major_, the *D*_supplementary_ is calculated on the condition that the NWs should support one mode for reducing reflection and material saving and their resonant wavelength should match the valley of the *D*_major_’s normalized absorption efficiency spectrum.

The periodicity of the NW arrays can be computed by construction of an effective medium layer. This artificial layer represents the reflection and transmission behavior of the NW arrays which is only related to the material FR. As a result, the diameter, periodicity, and the arrangement of the NW arrays are removed from the calculation. In this way, the transmittance and reflectance of NW arrays can be evaluated by applying Fresnel equations on this effective medium layer and therefore the optimal FR can be analyzed. Based on the relationship of FR and periodicity, the periodicities for both hexagonal and squarely arrangement NW arrays are obtained. Detailed description of our proposed method is presented in the following sections.

### A. Optimal Diameters of InP NW Arrays for Maximal Light Harvesting

To increase the light absorption, the number of resonant modes leading to strong absorption peaks should be maximized within the absorption region. On the blue end of the absorption region, incident AM 1.5G spectrum confines 300 nm as the high energy region. The critical wavelength *λ*_*c*_ of 925 nm (bandgap of InP 1.34 eV) limits the red end of the absorbing region. As a result, it is proved that the InP NWs that support two resonant modes locating inside the absorbing region are able to best improve the light absorption [29]. We expand this conclusion and use Mie theory to calculate the exact value.

According to the above conclusion, the range of *D*_major_ can be calculated from the eigenvalue equation derived from Maxwell’s equations [18]. Considering the anti-symmetric in-plane field distribution of the incident plane waves, only the HE_1m_ modes can be effectively excited to contribute to the absorption of vertically-aligned NWs [5]. These HE_1m_ modes satisfy the eigenvalue equation, and the resonant wavelengths can be obtained assuming that the real part of the propagation constant *Re(β*_*z*_ *)* of the mode along NW axial direction approaches zero as shown in Eq. (1). *k*_*cyl*_ and *k*_*air*_ are the transverse components of the wave vector inside the NWs and in the air whereas *ε*_*cyl*_ and *ε*_*air*_ are the respective permitivities. *J*_*1*_ and *H*_*1*_^*(1)*^ are the first order Bessel and Hankel functions of the first kind. As a consequence, the range that primary diameter falls in can be received on the condition that the corresponding HE_11_ and HE_12_ mode lie within the absorbing region.

1 $$ \frac{\varepsilon_{\mathrm{cyl}}{J}_1^{\prime}\left({k}_{\mathrm{cyl}}{D}_{\mathrm{major}}/2\right)}{k_{\mathrm{cyl}}{J}_1\left({k}_{\mathrm{cyl}}{D}_{\mathrm{major}}/2\right)}-\frac{\varepsilon_{\mathrm{air}}{H_1^{(1)}}^{\prime}\left({k}_{\mathrm{air}}{D}_{\mathrm{major}}/2\right)}{k_{\mathrm{air}}{H}_1^{(1)}\left({k}_{\mathrm{air}}{D}_{\mathrm{major}}/2\right)}=0. $$

According to the Mie theory, the absorption efficiency *Q*_abs_ of NWs is defined by the ratio of the energy collecting area and the geometrical size of the NWs. The analytical expression of absorption efficiency *Q*_abs_ is given below, and the exact mathematical formalism of Mie theory can be found in the reference [30]. Here, $\overline{n}=n+ ik$ is the complex refractive index; as mentioned above, *J*_*i*_ and *H*_*i*_^*(1)*^ are the Bessel and Hankel functions of first kind of order *i*.

2 $$ {\displaystyle \begin{array}{c}{Q}_{\mathrm{abs},\mathrm{TM}}=\frac{2}{x}\operatorname{Re}\left({b}_0+2\sum \limits_{i=1}^{\infty }{b}_i\right)-\frac{2}{x}\left[{\left|{b}_0\right|}^2+2\sum \limits_{i=1}^{\infty }{\left|{b}_i\right|}^2\right]\\ {}{Q}_{\mathrm{abs},\mathrm{TM}}=\frac{2}{x}\operatorname{Re}\left({a}_0+2\sum \limits_{i=1}^{\infty }{a}_i\right)-\frac{2}{x}\left[{\left|{a}_0\right|}^2+2\sum \limits_{i=1}^{\infty }{\left|{a}_i\right|}^2\right]\end{array}} $$

3 $$ {\displaystyle \begin{array}{c}{a}_i=\frac{\overrightarrow{n}{J}_i\left(\overrightarrow{n}x\right){J}_i^{\prime }(x)-{J}_i\left(\overrightarrow{n}x\right){J}_i^{\prime }(x)}{\overrightarrow{n}{J}_i\left(\overrightarrow{n}x\right){H_i^{(1)}}^{\prime }(x)-{J}_i^{\prime}\left(\overrightarrow{n}x\right){H}_i^{(1)}(x)}\\ {}{b}_i=\frac{J_i\left(\overrightarrow{n}x\right){J}_i^{\prime }(x)-\overrightarrow{n}{J}_i\left(\overrightarrow{n}x\right){J}_i^{\prime }(x)}{J_i\left(\overrightarrow{n}x\right){H_i^{(1)}}^{\prime }(x)-\overrightarrow{n}{J}_i^{\prime}\left(\overrightarrow{n}x\right){H}_i^{(1)}(x)}\end{array}} $$

After acquisition the *Q*_abs_ of the HE_11_ mode, the FWHM of respective diameter of NWs can be found out, and therefore, the optimal diameter for maximal light harvesting is determined. Upon decision of the major diameter, the supplementary diameter is confirmed on the condition that its normalized absorption peak wavelength should match the normalized absorption efficiency valley of the major diameter. For four diameter NW arrays, the third and fourth diameters are determined in a similar way. Their normalized absorption efficiency peaks should match the valleys of the superposition of normalized absorption efficiency spectrum of the primary and secondary NWs. It is noteworthy that except for the major NWs, the second, third, and fourth NWs are desired to support only one mode since the small diameter size can both reduce the reflectance at the air-NW interface and reduce material consumption.

### B. Optimal FR of InP NW Arrays for Maximal Light Harvesting

**Fig. 2:**
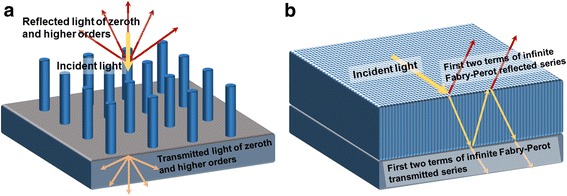
Light reflection, transmission, and absorption of NWs and effective medium layer. **a** InP NW arrays and **b** the corresponding effective medium layer with the same thickness

Various published work has disclosed that with fixed diameters of NWs; the absorption of the NWs will increase with the FR initially and then drop after a certain optimal value [13]. The rise of light absorption is usually attributed to the increase of volume percentage of the semiconductor materials with high absorption coefficients. As FR further grows, the average refractive index of the NW arrays increases, and thus, the reflection rises which reduces light absorption. Therefore, an upper limit on the FR should be found to optimize the influence of Fresnel reflection and transmission to maximize the absorption of the NW arrays. Figure 2 schematically illustrates that an effective medium layer of complex refractive index is created to represent the refraction and transmission behavior of the NW arrays. In this way, the periodicities and diameters of NWs are removed from the calculation. Consequently, Fresnel calculation of the reflection and transmission of the effective medium layer can be used to reflect the properties of the NW arrays. The exact nature inside this artificial medium layer is not considered as long as they can represent the reflection and transmission of NW arrays. Detailed mathematics derivations are given below.

The real part of the refractive index of the effective medium layer *n*_em_real_ are determined by Bruggeman formulation [31] in Eq. (4) where Ɛ_em_, and Ɛ_NW_ are the permittivity of the effective medium layer and InP, respectively. The imaginary part of the refractive index *n*_em_imag_ is calculated by Volume Averaging Theory [32, 33] in Eq. (5) where the *n*_NW_real_, *n*_NW_imag_, *n*_air_real_, and *n*_air_imag_ are the real and imaginary part of the refractive index of NW and air. The optimal FR_opt_ is defined as the FR such that absorptance *Abs(λ) = 1 − R(λ) − T(λ)* is maximized using Fresnel equations.

4 $$ {\displaystyle \begin{array}{l}\left(1-\mathrm{FR}\right)\frac{\varepsilon_{\mathrm{air}}^2-{\varepsilon}_{\mathrm{em}}^2}{\varepsilon_{\mathrm{air}}^2+2{\varepsilon}_{\mathrm{em}}^2}+\mathrm{FR}\frac{\varepsilon_{\mathrm{NW}}^2-{\varepsilon}_{\mathrm{em}}^2}{\varepsilon_{\mathrm{NW}}^2+2{\varepsilon}_{\mathrm{em}}^2}=0\\ {}{n}_{\mathrm{em}\_\mathrm{real}}=\operatorname{Re}\left(\sqrt{\varepsilon_{\mathrm{em}}}\right)\end{array}} $$

5 $$ {\displaystyle \begin{array}{l}\mathrm{A}=\mathrm{FR}\left({n}_{\mathrm{NW}\_\mathrm{real}}^2-{n}_{\mathrm{NW}\_\mathrm{imag}}^2\right)+\left(1-\mathrm{FR}\right)\left({n}_{\mathrm{air}\_\mathrm{real}}^2-{n}_{\mathrm{air}\_\mathrm{imag}}^2\right)\\ {}B=2\mathrm{FR}{n}_{\mathrm{NW}\_\mathrm{real}}{n}_{\mathrm{NW}\_\mathrm{imag}}+2\left(1-\mathrm{FR}\right){n}_{\mathrm{air}\_\mathrm{real}}{n}_{\mathrm{air}\_\mathrm{imag}}\\ {}{n}_{\mathrm{em}\_\mathrm{imag}}=\sqrt{\frac{-A+\sqrt{A^2+{B}^2}}{2}}\end{array}} $$

Through replacing the NW arrays with a thin film of equal thickness, the reflectance *R(λ)* and *T(λ)* transmittance of NW arrays can be estimated using the Fresnel equations. The first two terms of the infinite Fabry-Perot reflection and transmission series are included in Fig. 2b. Detailed mathematical derivations can also be found in the supporting information of the reference [13]. At this stage, the optimal diameters and the FR are both determined and the corresponding periodicity can be acquired based on the definition of the FR. With the optimal geometrical dimensions, the NW arrays should lead to maximal light absorption. Short-circuit current density *J*_sc_ is mostly used to measure the light harvesting capability assuming that every absorbed photon leads to an exciton separation followed by a successful carrier collection. The definition is shown in Eq. (6) where *A(λ)* is the absorption inside nanowires as a function of the incident wavelength, and *N(λ)* is the number of photons per unit area per second for the incident wavelength from the standard solar spectrum.

6 $$ {J}_{\mathrm{sc}}=q\underset{\mathrm{AM}1.5\mathrm{G}}{\int }A\left(\lambda \right)N\left(\lambda \right) d\lambda $$

## Results and Discussion

Single and multiple diameters of InP NW arrays of squarely and hexagonal arrangements demonstrate the validity of the proposed method. Meanwhile, FDTD numerical simulations (Lumerical FDTD Solutions 8.15) are also provided to compare with our method. Periodic boundary condition is applied along *x* and *y* axes while perfect matching condition is set along *z* axis as illustrated in Fig. 1. The InP NWs are vertically standing on SiO_2_ substrate. The optical constants for InP and SiO_2_ are from Palik material data provided by Lumerical. The parameter space for diameters of NWs ranges from 50 to 200 nm whereas the FR is from 0.05 to the possible maximal values for squarely and hexagonal NWs.

### A. Maximal Light Harvesting for Single Diameter InP NWs

**Fig. 3:**
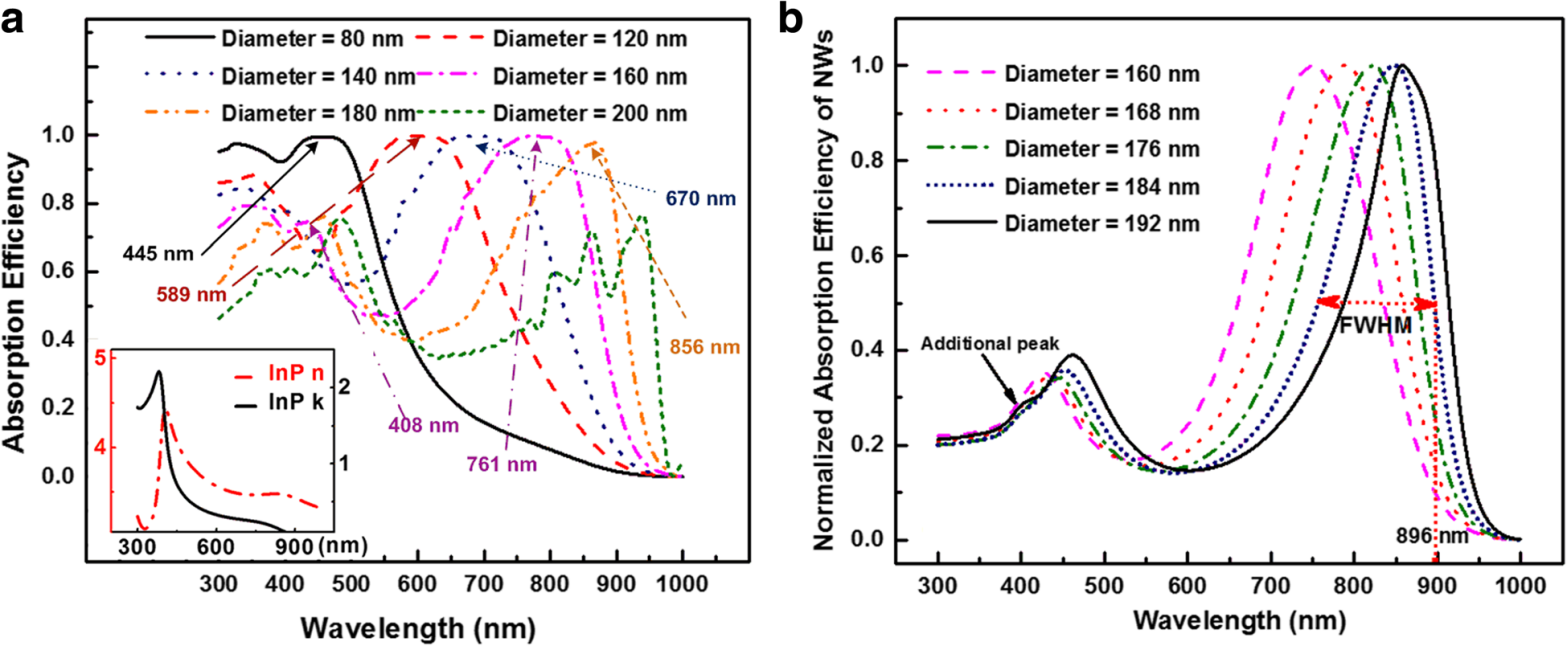
Wavelength dependent absorption efficiency of InP NWs and normalized absorption efficiency. **a** Absorption efficiency of NWs with inset explaining the optical constants. **b** Calculated absorption efficiency by Mie theory

Figure 3a shows the light absorption efficiency for single diameter InP NW arrays when FR is 0.05 with the optical constants provided in the inset. The respective resonant wavelengths are calculated and marked on corresponding absorbing peaks which match the FDTD simulation results well. The red shift of HE_11_ resonant mode can be easily observed with the rise of the diameter of NWs. Besides, both calculation and simulation prove that the resonant mode evolves from one to two modes at 140 nm diameter. Therefore, the optimal value for maximal light absorption should be larger than 140 nm and smaller than 200 nm where two modes are excited within each NW. To find the optimal value of diameter, normalized absorption efficiency of NW arrays is provided in Fig. 3b showing the NW arrays which support two modes and still keep the FWHM within the absorbing region. Therefore, the largest value of 184 nm diameter is chosen as the optimal diameter without any additional peak. Interestingly, the up-to-date highest power conversion efficiency InP NW solar cell design adopted the optimal diameter of 180 nm. Their diameters of NWs were experimentally optimized ranging from 50 to 300 nm with 10 nm as the increase step [9]. Compared with our prediction of 184 nm, a narrow tolerance of 4 nm demonstrates the accuracy of our method.

**Fig. 4:**
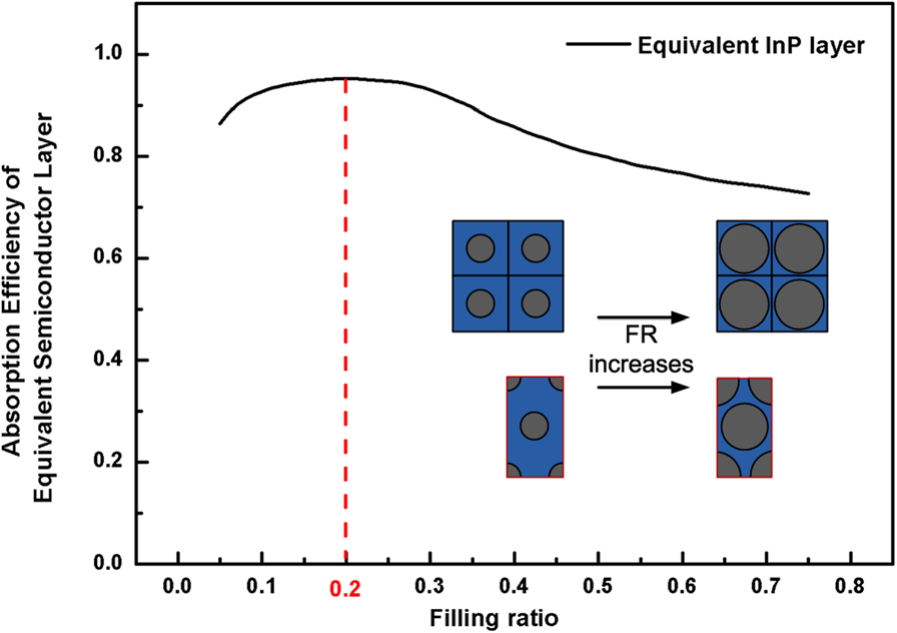
Absorption efficiency of effective medium layer for InP NW arrays as a function of FR

Filling ratio is analytically obtained using effective medium layer in section B of the described method. The light absorption efficiency of the effective layer of the same height as the InP NW arrays is shown in Fig. 4. In general, the light harvesting capability rises initially, reaches its maximal value, and gradually falls down as the FR approaches larger value. This trend is attributed to the change of the transmitted and reflected light as the complex refractive indices change due to FR variation. Specifically, when FR increases from 0.05 to 0.2, due to the addition of InP material, more light is absorbed before transmitted out of the NW arrays. However, this trend increase until FR reaches 0.2, and further increase of FR cause high complex refractive index of the equivalent layer which lead to optical impedance between the air and NW arrays. As a result, the reflectance at the incident surface rises rapidly which decrease light absorption [13]. Therefore, the optimal value for FR is 0.2 and the periodicities for squarely and hexagonal arranged NW arrays are 364.63 and 391.82 nm, respectively.

**Fig. 5:**
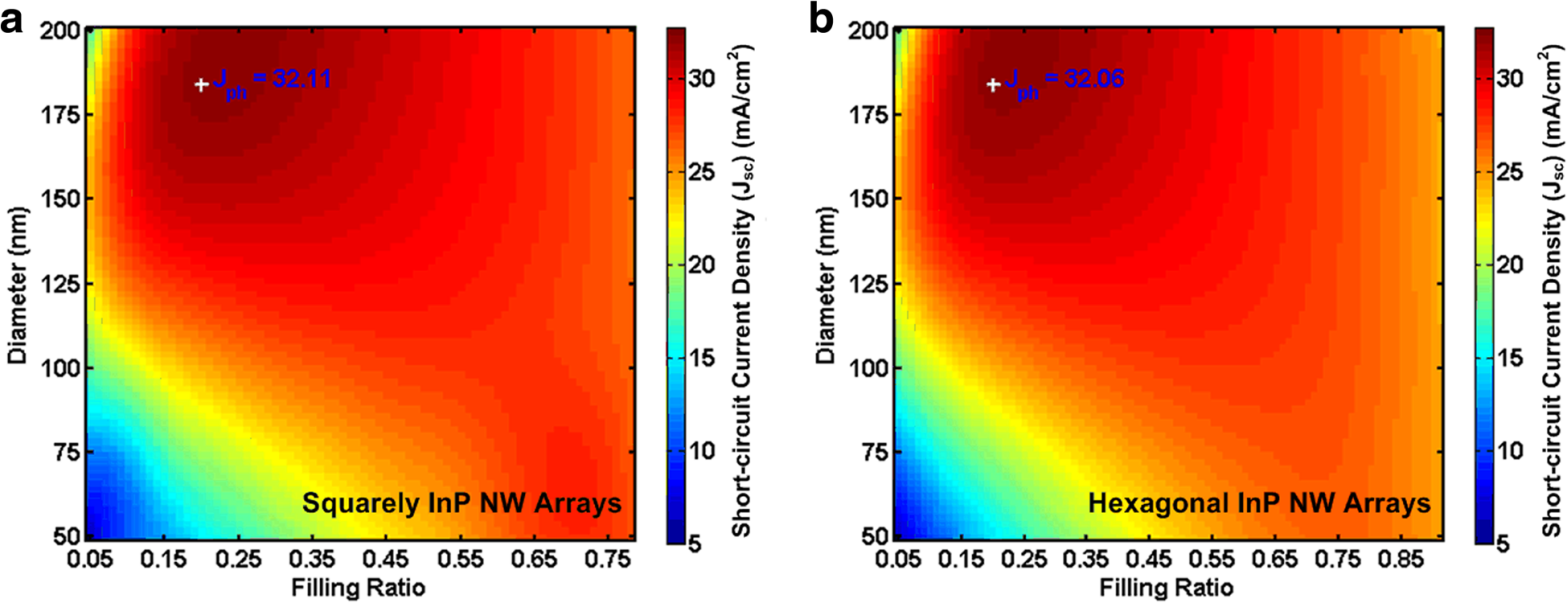
Theoretical predicted maximal values compared with FDTD simulations. **a** Squarely and **b** hexagonal single diameter InP NW arrays

The short-circuit current densities for various combinations of diameters and FRs are shown in Fig. 5. It clearly demonstrates that the arrangement of NWs has little effect on the highest light absorption. Also, regardless with the arrangements of NW arrays, our method can both be applied and achieve accurate results. The maximal *J*_sc_ with calculated optimal geometrical dimensions for InP NW arrays are calculated for squarely and hexagonal arrangement, respectively. The analytical predicted maximal *J*_sc_ is 32.11 and 32.06 mA/cm^2^ for squarely and hexagonal NW arrays leading to tolerance of 0.33 and 0.1%, respectively, as compared with FDTD simulation results.

### B. Maximal Light Harvesting for Double Diameter InP NWs

**Fig. 6:**
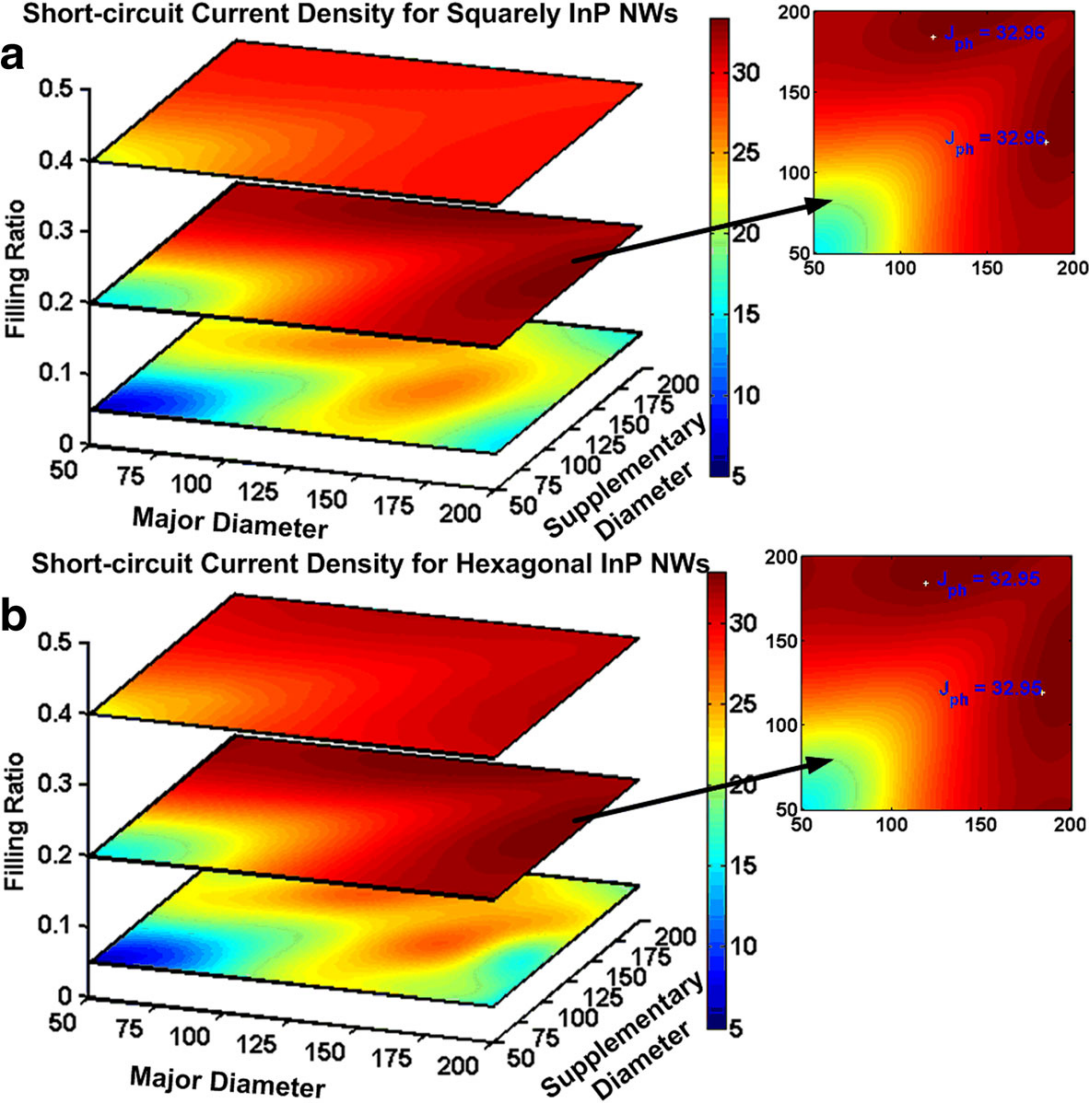
Short-circuit current densities as a function of major, supplementary diameters, and FRs. **a** Squarely and **b** hexagonal InP NW arrays where the insets show optimal diameters for respective NW arrangements

Adding a secondary diameter into the NW arrays has been investigated by several groups to further increase solar energy harvesting [22, 29] by time-consuming simulations [34]. From the above discussion, our method provides a way to fast approach the required NWs’ diameters. The resonant wavelength of the supplementary NWs should match the absorption valley of the major diameter of NWs which is 585 nm as shown in Fig. 3b. Also, the NWs should support only one resonant mode. These two conclusions lead to *D*_supplementary_ of 119 nm. The optimal FR of 0.2 still holds true in the double diameter InP NW arrays, and the periodicity is computed as 307 and 329.95 nm for squarely and hexagonal arrangement of NW arrays. Figure 6 provides an overview of the short-circuit current densities variation as a function of *D*_major_, *D*_supplementary_, and FR for two types of NW arrays. Generally, the light harvesting increases with FR, reaches its maximal value, and falls down. When FR is 0.2, the insets in Fig. 6 display the highest *J*_sc_ of 32.96 and 32.95 mA/cm^2^ for both squarely and hexagonal InP NWs. Compared with the maximal values from simulations as 33.34 and 33.26 mA/cm^2^, the tolerances are 1.1 and 0.9% for squarely and hexagonal NWs. Figure 6 also shows as FR grows, the coupling among neighboring NWs cannot be overlooked. Power can transfer to the neighboring NWs who support the same leaky mode causing the competition of the incident energy [35] which is detrimental to overall light absorption. When the FR is the same for both arrangements, p_square_^2^/p_hexagonal_^2^ is $\sqrt{3}/2$. Therefore, the *p*_hexagonal_ is 1.08 times of the *p*_square_ which has less mode coupling among NWs than square arrays. This explains the differences of the light harvesting of the two arrays when FR is 0.05 and 0.4.

### C. Maximal Light Harvesting for Four Diameter InP NWs

**Fig. 7:**
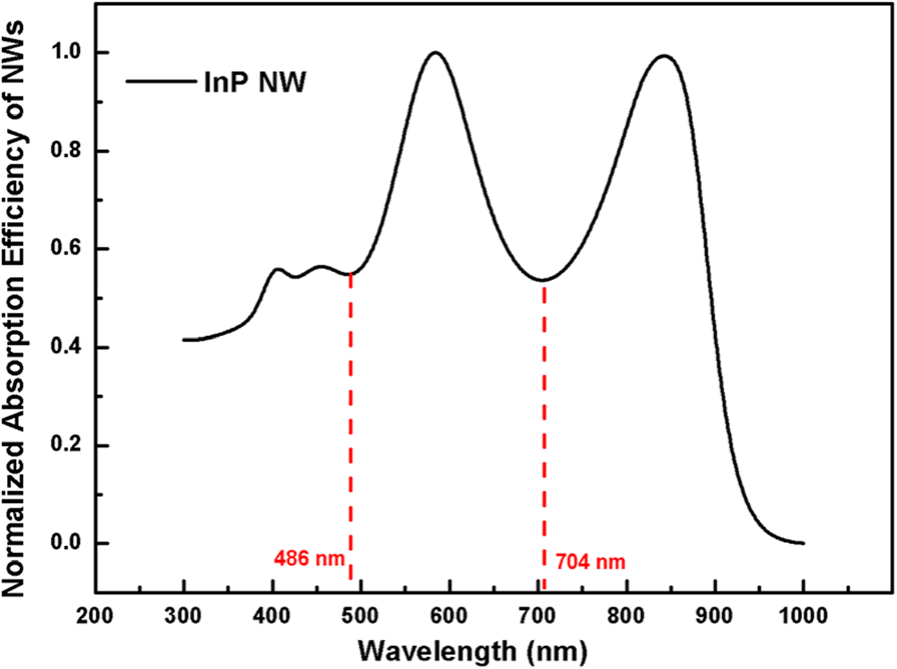
Superposition of the absorption efficiencies of the major and the supplementary diameters of InP NWs

Multiple diameters of NW arrays also attract lots of research interest to achieve near-unity absorption across the absorbing region [29]. However, only a limited number of diameter combinations are provided since the mass data acquisition requires large amount of time. This problem can be solved in our analytical design method, and four diameters of squarely arranged InP NW arrays are provided as an example. The total time taken to finish all of the calculations using our method equals to the time taken by only one FDTD simulation using the same personal computer. Upon acquisition of the major and supplementary diameters of NWs, the third and fourth diameters of NWs can be calculated in a similar way. The superposition of the normalized absorption efficiency of the major and supplementary diameter of NWs is shown in Fig. 7 with absorption valleys locating at 486 and 704 nm. Therefore, the third and fourth diameter of NWs can be computed to satisfy the conditions that each of them support only one mode, and the resonant wavelengths match the two absorption valleys in Fig. 7. Accordingly, the third and fourth diameters for InP NW arrays are obtained as 92 and 148 nm. With the optimal FR of 0.2 whose validity is irrespective of the arrangement of NW and diameters, the periodicity can be obtained as 277.41 nm for InP NW arrays.

**Fig. 8:**
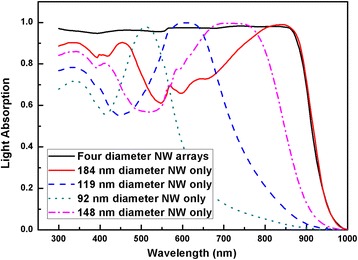
Light absorption of four diameter InP NW arrays

**Fig. 9:**
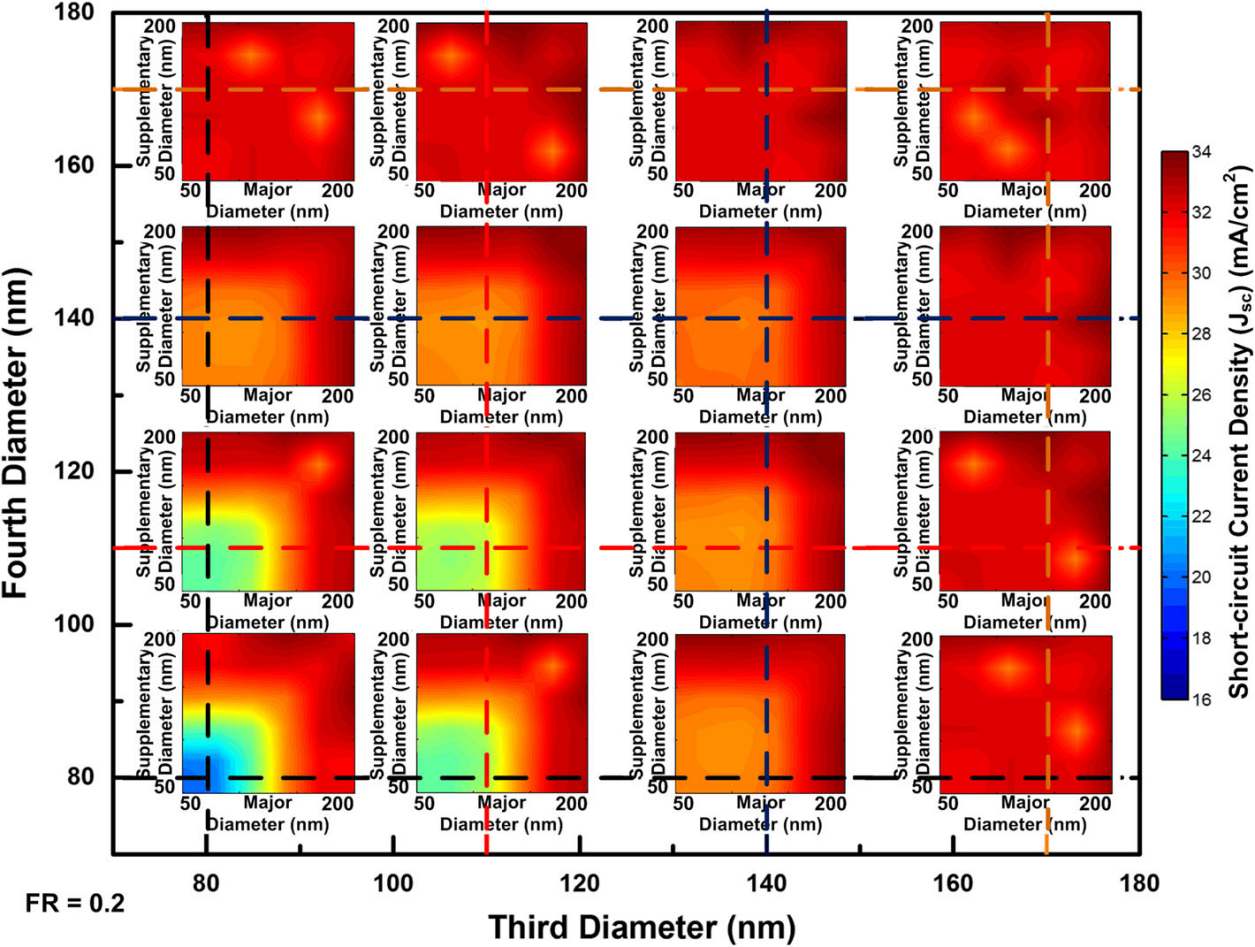
Short-circuit current densities change with the major, supplementary, third, and fourth InP NWs

The light absorption spectrum for the optimal combination of four NWs is provided in Fig. 8 from which the near-unity light absorption is achieved by the well selection of individual NWs. FDTD simulation results with four diameters’ combinations for squarely arranged NW arrays are shown in Fig. 9. To gain an overview of this multi-parameter optimization problem, two sets of coordinates are employed. The inner *x* and *y* axes denote the major and supplementary diameters whereas the outer *x* and *y* axes represent the third and fourth diameters. Due to the huge number of combinations of diameters, limited third and fourth diameters are deliberately selected to represent the whole absorption trend. From Fig. 3, the 80 nm is chosen as single mode resonance within NWs; 140 nm reflects the evolvement from single to double modes existence in NWs; 170 nm indicates the upper end of double modes existence while remain FWHM lying within absorbing region. Each intersect of the dash lines indicates different combination of the third and fourth diameters whereas the major and supplementary diameter run through 50 to 200 nm. When the diameters have larger values than 140 nm in Fig. 9, the majority of combinations of diameters will lead to the *J*_sc_ above 30 mA/cm^2^. When all of the diameters reach above 170 nm, the average of *J*_sc_ can be 32 mA/cm^2^. These results are also reflected in Figs. 5a and 6a. Compared with single or double diameter NW arrays, optimized four diameter NW arrays indeed lead to higher *J*_sc_. The highest *J*_sc_ for four diameters InP NW arrays with our calculated geometrical dimensions is 33.13 mA/cm^2^ with a tolerance of 2.2%.

## Conclusions

In this study, we present model for effective and fast design of both squarely and hexagonal InP NW arrays to achieve the highest light harvesting for photovoltaic application. Geometrical dimensions for vertically aligned single, double, and multiple diameters of NW arrays are investigated. Compared with time-consuming FDTD simulations, our predicted maximal short-circuit current densities with calculated three-dimensional NW arrays remain tolerances below 2.2% for all cases. For single diameter NW arrays, the optimal diameter is 184 nm which is only 4 nm difference to the reported highest efficiency InP NW solar cells. In the multiple diameter NW arrays, the diameters of the rest of NWs are optimized to satisfy the conditions that they support only one resonant mode and the corresponding wavelengths match the absorption valley of the major NWs. Moreover, the FR of the NW array is optimized to be 0.2 by creating an effective medium layer which is regardless of the diameter, periodicity, and arrangements of NWs. Compared with the optical modeling, the predicted highest short-circuit current densities for single diameter NW arrays lie within 0.33 and 0.1% tolerance for squarely and hexagonal NW array. The arrangements of NW array have little influence on the light absorption with optimal geometrical parameters, but the coupling among neighboring NWs becomes serious for multiple diameter NWs at large FR value. Squarely arranged four diameter NW arrays were also presented and the highest short-circuit current densities predicted to be 33.13 mA/cm^2^ with a low tolerance of 2.2%. The time-efficient, high precision with wide suitability of the proposed design for InP NW arrays demonstrate itself to be a promising tool to guide practical NW-based solar cell design.

## Abbreviations

FDTD: Finite-difference time-domain
FR: Filling ratio
FWHM: Full width at half maximum
NPs: Nanoparticles
NWs: Nanowires
